# Evidence for preferential attachment: Words that are more well connected in semantic networks are better at acquiring new links in paired-associate learning

**DOI:** 10.3758/s13423-020-01773-0

**Published:** 2020-07-07

**Authors:** Matthew H. C. Mak, Hope Twitchell

**Affiliations:** 1 Department of Experimental Psychology, Division of Medical Sciences, University of Oxford, Oxford, UK; 2 College of Behavioral & Social Sciences, Southeastern University, Lakeland, FL USA

**Keywords:** Preferential attachment, Semantic network, Degree centrality, Paired-associate learning (PAL), Adult free association

## Abstract

Here, we view the mental lexicon as a semantic network where words are connected if they are semantically related. Steyvers and Tenenbaum (*Cognitive Science, 29*, 41–78, 2005) proposed that the growth of semantic networks follows *preferential attachment*, the observation that new nodes are more likely to connect to preexisting nodes that are more well connected (i.e., the rich get richer). If this is the case, well-connected known words should be better at acquiring new links than poorly connected words. We tested this prediction in three paired-associate learning (PAL) experiments in which participants memorized arbitrary cue–response word pairs. We manipulated the semantic connectivity of the cue words, indexed by the words’ free associative degree centrality. Experiment 1 is a reanalysis of the PAL data from Qiu and Johns (*Psychonomic Bulletin & Review*, *27*, 114–121, 2020), in which young adults remembered 40 cue–response word pairs (e.g., *nature*–*chain*) and completed a cued recall task. Experiment 2 is a preregistered replication of Qiu and Johns. Experiment 3 addressed some limitations in Qiu and Johns’s design by using pseudowords as the response items (e.g., *boot*–*arruity*). The three experiments converged to show that cue words of higher degree centrality facilitated the recall/recognition of the response items, providing support for the notion that better-connected words have a greater ability to acquire new links (i.e., the rich do get richer). Importantly, while degree centrality consistently accounted for significant portions of variance in PAL accuracy, other psycholinguistic variables (e.g., concreteness, contextual diversity) did not, suggesting that degree centrality is a distinct variable that affects the ease of verbal associative learning.

Network science can be applied to any structure made up of nodes connected to each other through links (Hills, Maouene, Riordan, & Smith, 2010). For example, nodes might be people, and links might represent friendship or sexual contact. In recent years, network science has been applied to the study of complex cognitive systems, including the mental lexicon. Some of these studies (e.g., Griffiths, Steyvers, & Tenenbaum, 2007) view the mental lexicon as semantic networks, where word nodes are linked together by semantic relatedness. In their seminal paper, Steyvers and Tenenbaum (2005) reported that adult semantic networks possess structural properties that are believed to support efficient processing: sparse connectivity, short average path length, and strong local clustering. Importantly, the degree distribution of these semantic networks obeys power laws, such that the majority of words in the networks have few links to other words, but a minority of words serve as hubs, possessing links to many other word nodes. The emergence of a power-law distribution in networks is usually attributed to *preferential attachment*, a growth process by which the “rich get richer” (Barabasi & Alberts, 1999). Under this mechanism, new words are more likely to be learnt if they are linked to a known word with many preexisting connections (Castro & Siew, 2019). Alternative growth models have also been proposed; for instance, *preferential acquisition* (Hills, Maouene, Maouene, Sheya, & Smith, 2009) predicts that new words are more likely to be learnt if they are themselves well connected to other words in the learning environment, regardless of whether they are linked to a known word with many preexisting connections. ﻿It is, however, beyond the scope of the current article to offer an exhaustive comparison of these growth models; interested readers may consult these excellent articles: Beckage and Colunga (2019), Hills et al. (2009), and Fourtassi, Bian, and Frank (2019).

Assuming that adult semantic networks indeed follow a ﻿rich-get-richer growth pattern, it implies that well-connected words may have a stronger ability to acquire new links than poorly connected nodes (Mak, 2019). In other words, if a new link is attached to a well-connected word, this link may stand a higher chance of being learnt or remembered, compared with when it is attached to an isolated word node. Although this is a logical proposition, it is plausible that well-connected words may actually be *worse* at acquiring new links: Being well-connected means possessing more neighbors, which may compete for activation when the target word is activated (Hsiao, Mak, & Nation, 2019; Siew & Vitevitch, 2016, in press; Vitevitch & Stamer, 2006). New words, when linked to a well-connected word, may face more interference from its neighbors—in turn, reducing the chance of the new links being retained (Mak, 2019).

The current study set out to explore whether words higher in semantic connectivity are better at acquiring new links using paired-associate learning (PAL), a classic memory paradigm that assesses associative learning (Litt & Nation, 2014; ﻿Zaretsky & Halberstam, 1968). In a standard verbal PAL task, participants first memorize a list of arbitrary cue–response word pairs (e.g., *nature*–*chain*). At test, participants are provided with the cue words (e.g., *nature*) and are asked to recall the response words (e.g., *chain*) or to select the correct response words among a number of foils.

This article comprises three PAL studies: Experiment 1 is an exploratory analysis of the PAL data from Qiu and Johns (2020), in which young adults memorized 40 word pairs (e.g., *nature*–*chain*) and completed a cued recall task afterwards. Experiment 2 is a preregistered replication of Qiu and Johns (2020). Experiment 3 removed some potentially confounding variables in Qiu and Johns’s design by asking young adults to memorize word pairs made up of real and pseudowords (e.g., *boot*–*arruity*). These three experiments manipulated the semantic connectivity of the cue words, indexed by the words’ degree centrality in adult free association norms. The three experiments consistently demonstrated that cue words of higher degree centrality facilitated the recall and recognition of the response words, lending credence to the notion that well-connected words have a greater ability to acquire new links/associates. Importantly, while degree centrality consistently accounted for significant portions of variance in PAL accuracy in the data sets, other psycholinguistic variables (e.g., age of acquisition, concreteness, contextual diversity) did not, suggesting that degree centrality can capture something in verbal associative learning that other psycholinguistic metrics cannot.

## Calculating connectivity (i.e., degree centrality) in semantic networks

In a semantic network, each word is considered an individual node and is connected to other nodes via links that represent semantic relatedness, which can be operationalized in various ways: semantic associates generated in free association study, dictionary definitions, and semantic features (Hills et al., 2010). In the current study, we used adult free association norms (De Deyne, Navarro, Perfors, Brysbaert, & Storms, 2019)^1^ as the index of semantic relatedness, because (i) a majority of previous studies conceptualized semantic relatedness using such norms (e.g., Siew, 2019), and (ii) it has been shown to predict the order of early noun learning better than other semantic relatedness measures (e.g., Hills et al., 2009).

In De Deyne et al.’s (2019) free association norms, around 100 people gave three responses to a cue word (What are the first three words that came to mind upon seeing “dog”?), but for the sake of simplicity, only the first response was used here. Connectivity of a word is then indexed by the word’s degree centrality, which is the sum of its (i) out-degree and (ii) in-degree.

Out-degree refers to the number of distinct responses a cue word elicited. For example, the word *eruption* has an out-degree of 3 because it elicited three distinct first responses in the norming study: *volcanic*, *explosion*, and *volcano*.

In-degree, on the other hand, refers to how many times a word has been given as a first response. For example, *eruption* has an in-degree of 1, because only one cue word (e.g., *volcano*) elicited it as the first response. As such, the word *eruption* has a degree centrality of 3 + 1.

Note that in the calculation of both in-degree and out-degrees, we followed De Deyne and Storms (2008) and Nelson, McEvoy, and Schreiber (2004) in that a response is only considered “valid” if at least two persons in the norming study gave it as the response: For example, one participant (out of 98) gave *skincare* as a response upon seeing *eruption*. Such idiosyncrasy is unlikely to reflect true semantic relatedness, and hence, *skincare* was not counted towards the out-degree of *eruption*. Table 1 summarizes the descriptive statistics of degree centrality, and Table 2 shows the correlation values between degree centrality and other psycholinguistic metrics. As suggested by the reviewers and following previous studies (e.g., De Deyne, Navarro, & Storms, 2013; Steyvers & Tenenbaum, 2005), degree centrality was log (Base 10) transformed in all the statistical analyses ﻿to avoid the extreme positive skew inherent in degree distribution.

**Table 1.** Descriptive statistics of degree centrality of all the words sampled in De Deyne et al. (2019)

	Out-degree	In-degree	Degree centrality
Mean	11.9	10.8	22.7
Median	12	4	17
Standard deviation	3.64	23.6	24.2
Maximum	25	585	600
Minimum	1	0	1

*Note.* Total *N* of English words/phrases: 12,304

**Table 2.** Correlation values between (log-transformed) degree centrality and a range of psycholinguistic metrics

	1	2	3	4	5	6	7	8	Out-degree	In-degree
1. Degree centrality	1	–	–	–	–	–	–	–	0.23	0. 99
2. Frequency	.55	1	–	–	–	–	–	–		
3. Age of acquisition	−.48	−.40	1	–	–	–	–	–		
4. Concreteness	.06	.14	−.34	1	–	–	–	–		
5. Semantic diversity	.24	.48	−.44	−.17	1	–	–	–		
6. Contextual diversity	.62	.82	−.05	−.60	.46	1	–			
7. Phonological neighbor	.24	.26	.20	−.34	.07	.32	1			
8. Orthographic neighbor	.26	.24	.20	−.33	.06	.30	.81	1		

*Note. P* values of all correlations <.001. Total *N* of words: 12,304. Values for Metrics 2–8 were taken from the English Lexicon Project (Balota et al., 2007)

## Experiment 1: Exploratory analysis of Qiu and Johns (2020)

Although Qiu and Johns did not set out to investigate whether degree centrality of the cue words influenced recall performance in paired-associate learning, the cue words used in their study happened to possess suitable lexical properties that allow proper investigation into the matter.

First, there is good variation between the 40 cue words in terms of degree centrality (*Mdn* = 37.5, *SD* = 25.2, range: 13–124). Second, in this set of 40 cue words, most potentially confounding variables did not correlate with degree centrality: age of acquisition (*r* = −.17, *p* = .302), concreteness (*r* = −.10, *p* = .550), semantic diversity (*r* = .18, *p* = .266). Degree centrality, however, does correlate with log frequency among these 40 words (*r* = .56, *p* < .001), suggesting that log frequency, which is arguably the most recognized predictor in psycholinguistic tasks (Brysbaert, Mandera, & Keuleers, 2018), ought to be entered into the later statistical models so that its effect can be accounted for.

There were two data sets from Qiu and Johns: one from younger adults (18–29-year-olds) and one from older adults ﻿(45–60-year-olds). Only the former was reanalyzed here. This is because (i) Experiments 2 and 3 in the current study only recruited young adults, and (ii) PAL performance has been consistently shown to vary significantly across age groups (Rabbitt & Lowe, 2000).

Qiu and Johns (2020) reported that PAL performance of the younger participants was not affected by their experimental manipulation: semantic diversity of the cue words, a metric that ﻿quantifies the similarity of all the linguistic contexts a word appears in (e.g., Hoffman, Lambon Ralph, & Rogers, 2013; Hsiao & Nation, 2018; Johns, Dye, & Jones, 2016). In this reanalysis, we explored whether cue words that are better connected in semantic networks, indexed by degree centrality, facilitated the recall of the response words.

### Method

#### Participants

The data set has a sample size of 52 participants (*M*_age_= 23 years, *SD*_age_ = 3), recruited from Prolific.ac, an online subject pool for behavioral studies (Palan & Schitter, 2018). All participants reported to be native speakers of American English.

#### Materials

Forty English words varying in degree centrality [*M* = 44.7*, Mdn* = 37.5, *SD* = 25.2, range: 13–124, examples: *spring* (56), *lake* (38), *aunt* (17), *chin* (18)] served as the cue words. Another 40 English words (e.g., *chain*, *country*, *plate*, *soldier*) served as the response words. The cue and response words were matched on log frequency, imageability, and familiarity.

##### Pairing

A cue word was paired with a response word to form a word pair (e.g., *spring*–*chain*). The basis on which Qiu and Johns (2020) paired the words is as follows: 20 of the cue words (Cue List 1) could only be paired with 20 of the response words (Response List 1). Likewise, the remaining 20 cue words (Cue List 2) could only be paired with the remaining 20 response words (Response List 2). For every participant, the pairing was randomized, meaning that the maximum number of possible pairs was 800 (20 × 20 + 20 × 20). We checked the association strength of all these possible pairs, indexed by latent semantic analysis (LSA) cosine similarity (Landauer & Dumais, 1997). LSA cosines of these pairs were reasonably low (*M* = 0.10, *Mdn* = 0.07*, SD* = 0.10); this helped reduce the possibility that recall performance would be skewed by strong preexisting association strength.^2^

#### Procedure

There were four experimental blocks, each containing a learning phase and a cued recall test. In a learning phase, participants saw 10 different word pairs (e.g., *lake–soldier*), each presented individually on the computer screen for 1.5 seconds. Each pair was shown twice. Trial and block order were randomized.

Immediately after the learning phase, participants completed a cued recall test, in which the cue words were shown individually on the computer screen in a randomized order, and participants were asked to type out the response word they thought previously paired with the cue word. Following Qiu and Johns (2020), all spelling and typing errors were considered incorrect.

### Results

**Fig. 1:**
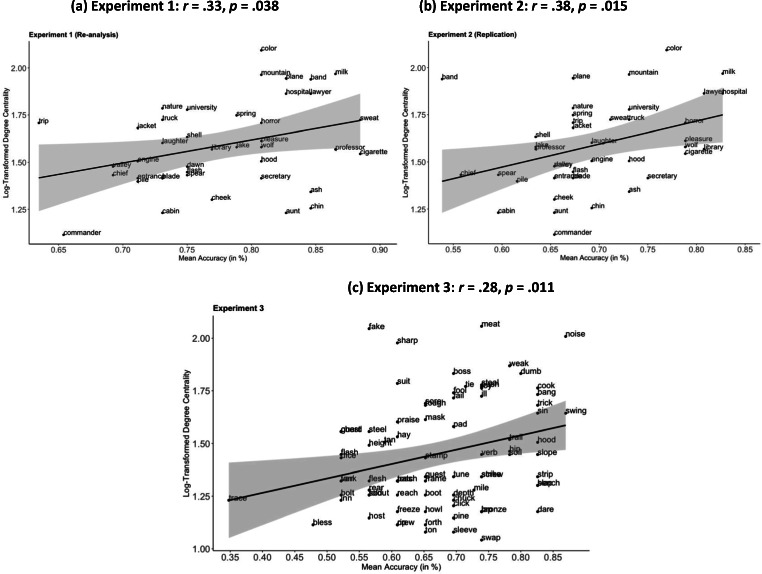
Scatterplots showing the correlations between degree centrality (of the cue words) and mean response accuracy in Experiments 1–3. Straight lines represent the best-fitting regression lines, and the shaded areas represent the standard error

We first examined the correlation between degree centrality of the cue words and the probability with which they elicited a correct recall. In line with the prediction of preferential attachment, degree centrality of the cue words was positively correlated with recall accuracy, *r*(38) = .33, *p* = .038. Figure 1a shows the scatterplot of this correlation.

Next, following Qiu and Johns (2020), the data were analyzed using a generalized linear mixed-effects model, computed using the lme4 package (Bates, Mächler, Bolker, & Walker, 2015) in R (Version 3.6.1; R Core Team, 2019). The dependent variable was accuracy (correct vs. incorrect), and the fixed factors were log-transformed degree centrality, log frequency, and their interaction. These fixed factors were entered as numerical variables and were scaled and centered. The model was computed following the procedure outlined in Jaeger (2011; see also Gaskell, Cairney, & Rodd, 2019). The models with the (near-)maximal random-effect structure either produced a singular fit or did not converge; therefore, we reported the model with random intercepts for participants and items only (see Table 3 for model summary). It showed that degree centrality (*b* = 0.25, *z* = 3.56, *p* < .001) and log frequency of the cue word (*b* = −0.19, *z* = −2.84, *p* = .005) both had a significant main effect on recall. Their interaction, however, was insignificant (*b* = 0.05, *z* = 0.72, *p* = .473).

**Table 3.** Model summary of the GLME models examining the effects of log-transformed degree centrality and log frequency on response accuracy in Experiments 1–3

	Experiment 1 (reanalysis)				Experiment 2 (replication)				Experiment 3			
	Estimate	*SE*	*z*	*p*	Estimate	*SE*	*z*	*p*	Estimate	*SE*	*z*	*p*
Intercept	1.51	0.17	9.01	<.001*	1.05	0.15	6.91	<.001*	0.77	0.09	8.45	< .001*
Log degree	0.25	0.07	3.56	<.001*	0.17	0.07	2.46	.014*	0.15	0.06	2.73	.006*
Log freq	−0.19	0.07	−2.84	.005*	−0.03	0.07	−0.39	.695	−0.09	0.06	−1.39	.166
Log degree × Log freq	0.05	0.06	0.72	.473	−0.08	0.06	−1.36	.175	0.03	0.07	0.40	.687

*Note*. Log degree = log-transformed degree centrality; Log freq = log frequency. *p < .05

#### Additional exploratory analyses

To further understand the effect of degree centrality on PAL, we checked whether the cue words’ age of acquisition (AoA), concreteness, semantic diversity, contextual diversity, and number of orthographic/phonological neighbors accounted for any variance in recall accuracy. Following reviewer’s advice, we compared fits of the degree*logFreq model with models that had a fixed effect of x*logFreq, where *x* was one of the said psycholinguistic variables (e.g., AoA). All the GLME models reported in this section were computed with the same procedure as before. The summaries of these additional models are shown in Table 4.

**Table 4.** Exploratory models examining the effects of a range of psycholinguistic variables, Experiment 1

	x =						
	Degree centrality	AoA	Concrete-ness	Semantic diversity	Contextual diversity	Orthographic neighbor	Phonological neighbor
x	*b =* 0.25 *z* = 3.56	*b =* 0.002 *z* = 0.03	*b =* 0.05 *z* = 0.65	*b =* −0.06 *z* = −0.84	*b =* 0.06 *z* = 0.74	*b =* 0.01 *z* = 0.17	*b =* −0.03 *z* = −0.38
Log freq	*b =* −0.19 *z* = −2.84	*b =* −0.06 *z* = −0.88	*b =* −0.06 *z* = −0.81	*b =* −0.05 *z* = −0.73	*b =* −0.10 *z* = −1.30	*b =* −0.05 *z* = −0.69	*b =* −0.06 *z* = −0.89
Interaction	*b =* 0.04 *z* = 0.72	*b =* −0.002 *z* = −0.05	*b =* 0.03 *z* = 0.27	*b =* −0.03 *z* = −0.47	*b =* −0.03 *z* = −0.43	*b =* 0.05 *z* = 0.61	*b =* 0.02 *z* = 0.33
AIC	1,984.9	1,996.6	1,995.9	1,995.5	1,995.5	1,996.1	1,996.3
BIC	2,018.8	2,030.4	2,029.7	2,029.4	2,029.3	2,030.0	2,030.1
Log likelihood	−986.5	−992.3	−991.9	−991.8	−991.7	−992.1	−992.1

The first point to highlight is that apart from degree centrality and log frequency, none of the potentially influential variables (e.g., AoA) came out as significant main effects (*p*s > .4). Among all the models, the one with degree*logFreq as the fixed effects had the smallest AIC/BIC values and the largest log-likelihood value (see lower portion of Table 4), suggestive of it having a better fit over its counterparts.

Finally, as requested by the reviewers, we explored (i) whether in-degree or out-degree of the cue words captured more variance in recall accuracy, and (ii) whether degree centrality and log frequency of the *response* words influenced recall accuracy.

For (i), we first checked the correlation between the in-degree and out-degree of the 40 cue words. The two measures did not correlate, *r*(38) < .01, *p* = .973. We then examined their effects on recall accuracy by entering in_degree*out_degree*logFreq as the fixed effects in a GLME model. In-degree had a significant main effect on recall accuracy (*b* = 0.26, *z* = 3.74, *p* < .001) while out-degree did not (*b* = 0.05, *z* = 0.65, *p* = .515).^3^ Note that the effect of in-degree appeared to be slightly greater than that of degree centrality (*b* = 0.25, *z* = 3.56, *p* < .001).

For (ii), we computed a GLME model with degree centrality and log frequency of the *response* words as the fixed factors (degree_response*freq_response). The model suggested that neither degree centrality nor log frequency of the *response* words had an effect on recall accuracy (degree_response: *b* = 0.06, *z* = 0.72*, p* = .475*;* freq_response: *b* = −0.09*, z* = *−*1.17*, p* = .241*;* interaction*: b* = −0.10*, z* = −1.33*, p* = .185).

### Discussion

The reanalysis of Qiu and Johns’s (2020) PAL data demonstrated that whether a response word was correctly recalled was influenced by the cue word’s degree centrality, which is taken as a proxy of how well connected the cue word is in preexisting semantic networks. Our reanalyses provided support for the hypothesis that well-connected words have a greater ability to acquire new links. Below, we outline two plausible accounts to explain why there is a tendency for “the rich to get richer.”

**Fig. 2:**
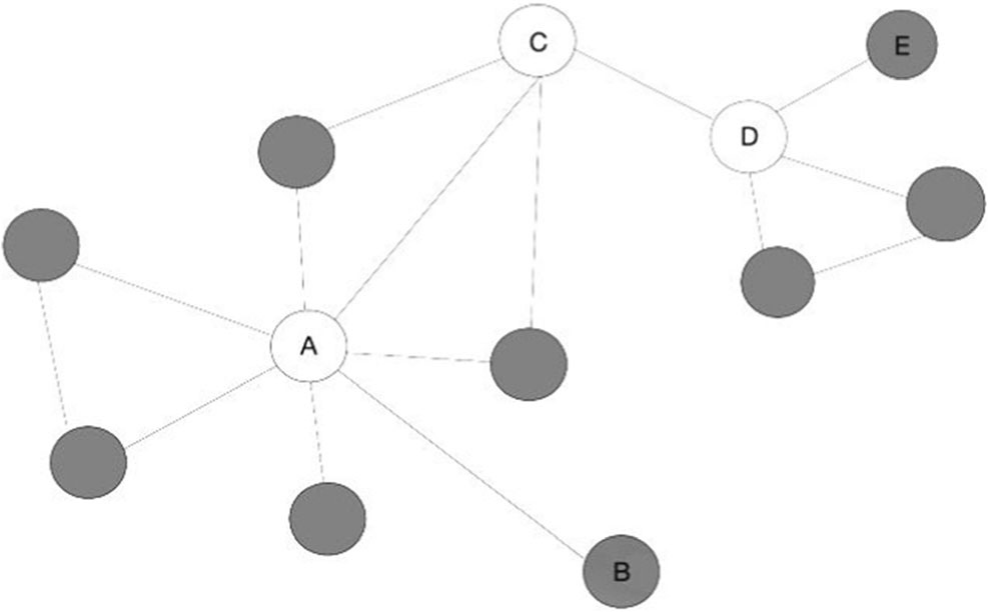
Illustration of why it may be easier for high-degree words to form arbitrary link with other words

First, high-degree (vs. low-degree) words are situated in more central locations in semantic networks (Steyvers & Tenenbaum, 2005), meaning that they are on average “closer” to all other words in the networks. Figure 2 illustrates this proposition.

In the network depicted in Fig. 2, the high-degree words (e.g., Nodes A, C, D) are in white, whereas the low-degree words (e.g., Nodes B & E) are in gray. The shortest path between a high-degree word and any other nodes in the network, on average, is relatively short. For instance, the average distance between Node A (a high-degree word) and all other nodes in Fig. 2 is 1.64 steps, whereas the average distance between Node B (a low-degree word) and all other nodes is 2.55 steps. This distance can be conceptualized as the amount of cognitive resources required to build an interitem association; the shorter the distance between two words, the easier it is to form and retain an arbitrary association between them. Since high-degree cue words are generally closer to all other words in semantic networks (Steyvers & Tenenbaum, 2005), it is not surprising that they facilitated PAL performance in Experiment 1.

There is, however, another equally plausible explanation. Previous studies have consistently shown that high-degree (vs. low-degree) words have a tendency to co-occur with many other words in natural languages, and they tend to appear in a wide range of speech and contexts (Fourtassi et al., 2019; ﻿Fourtassi & Dupoux, 2013; Hills et al., 2010; note also the moderate correlation, *r* = .62, between contextual diversity and degree centrality in Table 2). High-degree words, given their rich contextual history, may have grown to be more flexible and context-independent, and hence better placed to form associations with other words (see also Mak, Hsiao, & Nation, 2020a). It is worth noting that words high in *semantic* or *contextual diversity* (Adelman, Brown, & Quesada, 2006; Hoffman et al., 2013) also have rich contextual history, but, interestingly, these corpus-derived metrics did not seem to account for any variance in PAL performance in Experiment 1. This suggests that the kind of contextual history captured by semantic/contextual diversity may be different from that encoded in degree centrality. While the contextual history captured by the former is primarily linguistic in nature, we speculate that degree centrality may reflect a word’s contextual history that encompasses not only its linguistic but also its spatial and temporal usage. Consider the analysis on the effect of in-degree versus out-degree, which showed that in-degree captured substantially more variance in recall accuracy. In-degree is positively correlated with lexical availability (De Deyne et al., 2013), suggesting that words of higher in-degree are more likely to be used by a person in a wider range of documents, social situations, and time of day. It might be all these varieties in linguistic/spatial/temporal contexts that have made high-degree words flexible and context-independent, and hence better placed to form arbitrary associations. This would then explain why degree centrality, but not semantic/contextual diversity, showed an effect on PAL accuracy. Future work using computational and big data approaches is needed to examine our speculation (see, e.g., Roy, Frank, DeCamp, Miller, & Roy, 2015).

Before moving on, let us consider the in-degree versus out-degree analysis once more. The degree centrality effect found in Experiment 1 was driven by in-degree instead of out-degree (see also De Deyne et al., 2013). Notably, lexical growth models based on preferential attachment (e.g., Steyvers & Tenenbaum, 2005) predict that well-connected words are more likely to acquire new links, but these models did not specify whether well-connectedness refers to in-degree, out-degree, or degree centrality. The finding that in-degree explained more variance in PAL accuracy than out-degree and even degree centrality suggests that the notion of well-connectedness may be better conceptualized in terms of in-degree. In light of this, we considered switching our focus from degree centrality to in-degree in the subsequent experiments, reported later in this paper; however, the analysis of in-degree versus out-degree was motivated by a reviewer’s suggestion, and the remaining experiments did not set out a priori to investigate in-degree, so we kept our focus on degree centrality in the subsequent experiments.

Finally, we also explored whether the cue words’ age of acquisition, concreteness, and number of orthographic/phonological neighbors explained any variance in PAL performance. None of them appeared to be significant predictors. We also confirmed that degree centrality and log frequency of the *response* words did not have a significant effect on recall accuracy. All these suggest that the degree centrality effect in Experiment 1 was unlikely to be attributable to a third factor. However, it is important to stress that our reanalyses were exploratory in nature, so the results should be interpreted with caution (Bishop, 2020; Grove & Andreasen, 1982). In light of this, we replicated Qiu and Johns (2020) in a confirmatory study. This replication was preregistered ahead of data collection, and the preregistration document is available at https://osf.io/7vejf.

## Experiment 2: Preregistered replication of Qiu and Johns (2020)

### Method

#### Participants

A total of 55 young adults (11 males, *M*_age_ = 24.4 years, *SD*_age_ = 3.4) were recruited from Prolific.ac. All reported to be native speakers of English and having no history of any language/learning disorders. Three of the participants were excluded from further analyses, as they failed more than one attention check. As in Qiu and Johns (2020), the final sample size was 52.

#### Procedure

Experiment 2 used the same stimuli and procedure as Qiu and Johns (2020). There was, however, one difference with regard to how the cue and response words were paired. In Qiu and Johns, the maximum number of possible pairs was 800. In this replication study, the number was doubled to 1,600 because each of the 40 cue words now had an equal probability to be paired with each of the 40 response words. This change was to further reduce the potential influence of any preexisting association strength on recall accuracy. LSA cosines of these 1,600 possible pairs (*M* = 0.10, *Mdn* = 0.07*, SD* = 0.10) were as low as the 800 pairs in Experiment 1.

### Results

As per Experiment 1, we first examined the correlation between degree centrality of the cue words and the probability with which they elicited a correct response. Figure 1b shows the scatterplot of this correlation. In line with Experiment 1, degree centrality of the cue words was positively correlated with recall accuracy, *r*(38) = .38, *p* = .015.

#### Confirmatory analyses

A generalized linear mixed-effects model, computed using the same procedure as before, showed that degree centrality had a significant main effect on recall accuracy (*b* = 0.17, *z* = 2.46, *p* = .014). The main effect of log frequency, in contrast to Experiment 1, was insignificant here (*b* = −0.03, *z* = −0.39, *p* = .695). Their interaction also failed to reach statistical significance (*b* = −0.08, *z* = −1.36, *p* = .175; for model summary, see Table 3).

#### Exploratory analyses

We performed the same additional exploratory analyses as before. The results mirrored those in Experiment 1, confirming that (i) including degree centrality as a fixed effect resulted in the best model fit, suggesting that it can capture something in verbal associative learning that other psycholinguistic variables (AoA, contextual diversity) cannot, and (ii) in-degree captured more variance in recall accuracy than out-degree. Details of these additional analyses, due to space limitations, are not reported here, but are available in full on the Open Science Framework (http://osf.io/7942s).

Of note here, though, is that, in contrast to Experiment 1, degree centrality and log frequency of the *response* words both had a significant main effect on recall accuracy in the current replication (degree_response: *b* = 0.12, *z* = 2.05, *p* = .040; freq_response: *b* = −0.12, *z* = −2.11, *p* = .034; interaction: *b* = −0.09, *z* = −1.47, *p* = .140). This prompted a further analysis that added degree centrality of the *cue* word into the model (degree_response*freq_response*degree_cue). In line with the confirmatory analysis, degree centrality of the *cue* words remained a significant predictor (degree_cue: *b =* 0.18, *z* = 2.85, *p =* .004), and its inclusion also improved model fit, as indicated by AIC/BIC/log likelihood values (full model available on the Open Science Framework).

### Discussion

Experiment 2 replicated the main finding of Experiment 1: Response words were more likely to be recalled if they were previously paired with a cue word higher in degree centrality. This provides credence for adult semantic networks growing in accordance with the principle of preferential attachment, which predicts that well-connected words have a stronger ability to acquire new links.

Reiterating briefly here, high-degree cue words might facilitate PAL via two mechanisms. The first one is that these words, since they occupy more central locations in semantic networks, are generally closer to all other words. This might facilitate the ease with which arbitrary associations are formed and retained. The second mechanism is that high-degree words are better placed to form arbitrary associations because they have been used and experienced in more diverse linguistic/spatial/temporal contexts. These two proposed mechanisms are not mutually exclusive and may work in tandem to bring about the facilitative effect seen in Experiments 1 and 2.

Nevertheless, there are two intrinsic issues with the current experimental design. First, the number of cue words were fixed at 40. It is not clear whether the effect of degree centrality found in Experiments 1 and 2 was stimulus specific. Therefore, it was necessary to adopt an expanded and different word set as cue words to ensure that the effect is generalizable. Second, if high-degree cue words indeed have a stronger ability to acquire new links as predicted by preferential attachment, this effect should extend to when pseudowords (e.g., *arruity*) are used as the response items. An advantage of using pseudowords as the response items is that they have no psycholinguistic baggage, allowing us to obtain a cleaner picture. If high-degree cue words can boost the learning and retention of pseudo response words, it would provide strong support for the account that well-connected words, due to their rich contextual history, have grown to be better placed to form arbitrary associations. Taking all these into considerations, we decided to double the size of the cue word set (*N* = 80) in Experiment 3 and ask participants to memorize word pairs in which the response items were pseudowords (e.g., *boot*–*arruity*).

## Experiment 3

### Method

#### Participants

A total of 50 participants (16 males, *M*_age_ = 19.7 years, *SD*_age_ = 3.3) from the University of Oxford took part in the study for payment or partial course credits. Four were excluded from further analyses as they had dyslexia (*N* = 1) or did not speak English as their first language (*N* = 3). All subsequent analyses were therefore based on the remaining 46 participants.

#### Materials

A total of 80 monosyllabic words were chosen from Hoffman and Woollams (2015) to serve as the cue words [e.g., high-degree: *Mdn* = 48, *SD* = 22.8, range: 27–114, examples: *chest* (36), *fool* (55), *boss* (68). Low-degree: *Mdn* = 18, *SD* = 3.4, range: 11–22, examples: *ton* (12), *rip* (13), *bench* (20)].^4^ The high-degree and low-degree words were matched on log frequency, *t*(70.4) = −0.18, *p* = .85, and age of acquisition, *t*(71.9) = −1.52, *p* = .13. The 80 words were then divided into two lists. Participants were randomly assigned to learn either one of the lists.

The response words were 40 seven-letter pseudowords (e.g., *arruity*, *clartle*, *dradden*, *slimble*), chosen from the English Lexicon Project (Balota et al., 2007). They had no orthographic neighbors and were similar in terms of bigram frequency.

#### Procedure

Experiment 3 started with a learning phase, followed by a two-alternative forced-choice (2AFC) test. In the learning phase, participants were asked to memorize 40 word pairs, each consisting of a real word (e.g., *boot*) and a pseudoword (e.g., *arruity*). The pairing was randomized. Each pair was shown individually on the computer screen for 4 seconds, and each pair was seen twice. Given the novelty of the pseudowords, we found it necessary to promote *active* learning, so for half of the trials, participants were asked to recall the word pair immediately after it was shown (i.e., type in the word pair you just saw). Each pair was recalled once in the learning phase, and trial order was randomized.

In the subsequent 2AFC test, each trial presented a real word from the learning phase (e.g., *boot*), along with two pseudowords that were also encountered in the learning phase (e.g., *arruity*, *clartle*). Participants were asked to decide which of the two options was previously paired with the real word. Trial order was randomized. The use of the 2AFC test here diverged from Experiments 1 and 2 where cued recall was used to tap PAL. Initially, we wished to use cued recall in Experiment 3 as well, but a pilot study clearly demonstrated a floor effect in the recall of pseudowords.

### Results

Due to oversight, an item (i.e., *ridge*) was used as a cue word even though it was not sampled in De Deyne et al. (2019; i.e., no data about its degree centrality were available). This item was therefore removed from further analyses. In addition, 14 data points (0.77% of all data points) were lost due to a programming error. The final analysis was based on 1,803 data points from 46 participants.

As before, we first examined the correlation between a cue word’s degree centrality and the probability with which it elicited a correct response. In line with the previous experiments, degree centrality and response accuracy were positively correlated, *r*(77) = .28, *p =* .011 (for scatterplot, see Fig. 1c).

Then, we followed in the footsteps of Experiments 1 and 2 by examining the effects of (log-transformed) degree centrality and log frequency in a generalized linear mixed-effects model, computed and selected using the same procedure as before. The model showed that degree centrality had a significant main effect on response accuracy (*b* = 0.15, *z* = 2.73, *p* = .006), while log frequency did not (*b* = −0.08, *z* = −1.39, *p* = .403). Their interaction was also insignificant (*b* = 0.03, *z* = 0.40, *p* = .687).

## General discussion

In three paired-associate learning (PAL) experiments, we tested whether words that are more well connected in semantic networks have a stronger ability to acquire new links. Connectivity was indexed by the words’ degree centrality derived from adult free association norms (De Deyne et al., 2019). The three experiments converged to show that the response items were more likely to be recalled and recognized if they were previously paired with a cue word that was higher in degree centrality (i.e., more well connected). This lends credence to the notion that the adult lexicon grows following the principle of preferential attachment.

Among the three studies reported here, Experiment 3 provided particularly compelling evidence for the more-gets-more growth pattern. In Experiments 1 and 2, the *response* words were real words (e.g., *chain*); even though effort was made to minimize their potential influence on PAL, it remained possible that their psycholinguistic properties modulated the effect of degree centrality. This possibility, however, was eliminated in Experiment 3, where the response items were neighbor-less pseudowords (e.g., *arruity*). In addition, Experiment 3 used an expanded set of cue words (*N* = 80), whose characteristics were also better controlled (e.g., all cue words were monosyllabic; high-degree and low-degree cue words were matched on frequency and AoA). In sum, Experiment 3 confirmed that the effect of degree centrality on PAL is not stimulus specific and is generalizable.

We proposed two mechanisms to account for why “the rich were able to get richer.” The first one is that high-degree words are located in more central locations in semantic networks, meaning that they are in greater proximity to all other words in the networks. This account, however, cannot explain the finding in Experiment 3, as the response items were pseudowords that had no preexisting representations in the mental lexicon. The alternative account, on the other hand, sits comfortably with the data from the three experiments: It postulated that high-degree words, as a result of them having been used and experienced in more diverse linguistic/spatial/temporal contexts, have become more flexible and context-independent, and hence better placed to form arbitrary associations with other items. Of relevance here is the notion of *mutability*, which refers to a word’s propensity to take on different senses across linguistic contexts (Asmuth & Gentner, 2017; Gentner & Asmuth, 2019). Since high-degree words tend to appear in more diverse linguistic contexts, they may also be more polysemous or mutable in meaning. This might have contributed to their greater flexibility in forming arbitrary associations with other items (see also Bowdle & Gentner, 2005). Future work is needed to investigate (i) the extent to which degree centrality (a continuous measure) is related to the notion of mutability (a discrete concept in Asmuth & Gentner, 2017) and (ii) what contextual history (e.g., linguistic/spatial/temporal) is encoded in degree centrality, as opposed to the kind reflected by semantic/contextual diversity.

Previous studies have examined the effect of degree centrality in single-word processing, showing that high-degree words are advantaged in visual lexical decision (Balota, Cortese, Sergent-Marshall, Spieler, Yap, 2004; De Deyne et al., 2013; Steyvers & Tenenbaum, 2005), but disadvantaged in free recall (e.g., Nelson, Bennett, Gee, Schreiber, McKinney, 1993; Nelson & Schreiber, 1992). The current study, as far as we are aware, is the first to investigate how the degree centrality of one word affects the learning of *another* word, and the results here show promise that degree centrality is a valid psycholinguistic variable that affects verbal associative learning. Future work may consider using other paradigms, such as serial recall, to further understand the role of degree centrality in associative learning and whether it may be modulated by for example task demand. Indeed, ongoing work in the first author’s lab seems to align very well with the PAL data presented here: In six separate serial recall data sets, words higher in degree centrality were recalled more accurately, across serial positions and independently of frequency (Mak, Hsiao, & Nation, 2020b). This, alongside the current set of experiments, reinforces the idea that high-degree words are better at forming new and arbitrary associations with other words.

Finally, one may wonder why we used degree centrality but not other connectivity measures (e.g., clustering coefficients, PageRank; for a review, see Siew, Wulff, Beckage, & Kenett, 2019). The reasons are twofold: First, degree centrality is arguably the most important connectivity measure in any network. Second, it is straightforward. Future work should explore whether other network measures influence associative learning and whether it is worthwhile to incorporate weightedness into the calculation of degree centrality (see, e.g., De Deyne et al., 2013).

### Open practices statement

All materials, data, analysis scripts, and exploratory analyses not reported here because of space limitations are publicly available at http://osf.io/7942s.

## Footnotes

1 We used the free associate norms by De Deyne et al. (2019) instead of the one by Nelson, Evoy, and Schreiber (2004) because the former is more recent and sampled more words.

2 The LSA cosine between a pair of words has been shown to influence the ease with which an association is formed (e.g., Tse & Altarriba, 2007). An exploratory generalized linear mixed-effect model with LSA cosine as the sole fixed factor showed that it did not predict recall accuracy in either Experiment 1 (*b* = 0.49, *z* = 0.77, *p* = .429) or Experiment 2 (*b* = 0.49, *z* = 0.87, *p* = .384). This null effect is probably because there was little variation in LSA cosines among the word pairs.

3 Log frequency remained a significant main effect (*z* = −3.18, *p* = .001) here, and none of the interaction terms came out statistically significant (*p*s *>* .2).

4 One reviewer noted that in De Deyne et al.’s (2019) free association norms, the median degree centrality is 17, suggesting that words with a degree centrality of, for example, 20, are not actual “low-degree” words. However, since we needed to control for frequency, age of acquisition, and number of syllables, restricting ourselves to words with a degree centrality of <17 proved to be very difficult. We therefore had to relax the upper limit to 22 when selecting the “low-degree” words.

## References

[CR1] Adelman, J. S., Brown, G. D. A., & Quesada, J. F. (2006). Contextual diversity, not word frequency, determines word-naming and lexical decision times. *Psychological Science, 17*, 814–823. 10.1111/j.1467-9280.2006.01787.x 10.1111/j.1467-9280.2006.01787.x 16984300

[CR2] Asmuth J, Gentner D (2017). Relational categories are more mutable than entity categories. Quarterly Journal of Experimental Psychology.

[CR3] Balota DA, Cortese MJ, Sergent-Marshall SD, Spieler DH, Yap MJ (2004). Visual word recognition of single-syllable words. Journal of Experimental Psychology: General.

[CR4] Balota DA, Yap MJ, Hutchison KA, Cortese MJ, Kessler B, Loftis B (2007). The English Lexicon Project. Behavior Research Methods.

[CR5] Barabasi AL, Albert R (1999). Emergence of scaling in random networks. Science.

[CR6] Bates D, Mächler M, Bolker B, Walker S (2015). Fitting linear mixed-effects models using lme4. Journal of Statistical Software.

[CR7] Beckage NM, Colunga E (2019). Network growth modeling to capture individual lexical learning. Complexity.

[CR8] Bishop DVM (2020). The psychology of experimental psychologists: Overcoming cognitive constraints to improve research: The 47th Sir Frederic Bartlett Lecture. Quarterly Journal of Experimental Psychology.

[CR9] Bowdle BF, Gentner D (2005). The career of metaphor. Psychological Review.

[CR10] Brysbaert M, Mandera P, Keuleers E (2018). The word frequency effect in word processing: An updated review. Current Directions in Psychological Science.

[CR11] Castro, N., & Siew, C. S. Q. (2019). *Contributions of modern network science to the cognitive sciences: Revisiting research spirals of representation and process*. 10.31234/osf.io/gkmb8 10.1098/rspa.2019.0825 PMC7428042 32831584

[CR12] De Deyne S, Navarro DJ, Perfors A, Brysbaert M, Storms G (2019). The “Small World of Words” English word association norms for over 12,000 cue words. Behavior Research Methods.

[CR13] De Deyne S, Navarro DJ, Storms G (2013). Better explanations of lexical and semantic cognition using networks derived from continued rather than single-word associations. Behavior Research Methods.

[CR14] De Deyne S, Storms G (2008). Word associations: Network and semantic properties. Behavior Research Methods.

[CR15] ﻿Fourtassi, A., Bian, Y., & Frank, M. C. (2019). *The growth of children’s semantic and phonological networks: Insight from 10 languages*. 10.31234/osf.io/37npj 10.1111/cogs.12847 32621305

[CR16] Fourtassi, A., & Dupoux, E. (2013). A corpus-based evaluation method for distributional semantic models. *51st Annual Meeting of the Association for Computational Linguistics Proceedings of the Student Research Workshop* (pp. 165–171). Retrieved from https://www.aclweb.org/anthology/P13-3024/

[CR17] Gaskell MG, Cairney SA, Rodd JM (2019). Contextual priming of word meanings is stabilized over sleep. Cognition.

[CR18] Gentner D, Asmuth J (2019). Metaphoric extension, relational categories, and abstraction. Language, Cognition and Neuroscience.

[CR19] Griffiths TL, Steyvers M, Tenenbaum JB (2007). Topics in semantic representation. Psychological Review.

[CR20] Grove WM, Andreasen NC (1982). Simultaneous tests of many hypotheses in exploratory research. Journal of Nervous and Mental Disease.

[CR21] Hills TT, Maouene M, Maouene J, Sheya A, Smith L (2009). Categorical structure among shared features in networks of early-learned nouns. Cognition.

[CR22] Hills TT, Maouene J, Riordan B, Smith LB (2010). The associative structure of language: Contextual diversity in early word learning. Journal of Memory & Language.

[CR23] Hoffman P, Lambon Ralph MA, Rogers TT (2013). Semantic diversity: A measure of semantic ambiguity based on variability in the contextual usage of words. Behavior Research Methods.

[CR24] Hoffman P, Woollams AM (2015). Opposing effects of semantic diversity in lexical and semantic relatedness decisions. Journal of Experimental Psychology: Human Perception and Performance.

[CR25] Hsiao, Y., Mak, M. H. C., & Nation, K. (2019). *The influence of semantic diversity on serial recall of words.* Poster presented at the 41st Annual Conference of the Cognitive Science Society. Montreal, Quebec, Canada.

[CR26] Hsiao Y, Nation K (2018). Semantic diversity, frequency and the development of lexical quality in children’s word reading. Journal of Memory and Language.

[CR27] Jaeger, T. F. (2011). More on random slopes and what it means if your effect is not longer significant after the inclusion of random slopes [Blog post]. Retrieved from https://hlplab.wordpress.com/2011/06/25/more-on-random-slopes/

[CR28] Johns BT, Dye M, Jones MN (2016). The influence of contextual diversity on word learning. Psychonomic Bulletin & Review.

[CR29] Landauer TK, Dumais ST (1997). A solution to Plato’s problem: The latent semantic analysis theory of acquisition, induction and representation of knowledge. Psychological Review.

[CR30] Litt RA, Nation K (2014). The nature and specificity of paired associate learning deficits in children with dyslexia. Journal of Memory and Language.

[CR31] Mak, M. H. C. (2019). Why and how the co-occurring familiar object matters in Fast Mapping (FM)? Insights from computational models. *Cognitive Neuroscience*, *10*(4), 229–231. 10.1080/17588928.2019.1593121 10.1080/17588928.2019.1593121 30894067

[CR32] Mak, M. H. C., Hsiao, Y., & Nation, K. (2020a). *Anchoring and contextual variation in the early stages of incidental word learning during reading*. 10.31219/osf.io/kf96e

[CR33] Mak, M. H. C., Hsiao, Y., & Nation, K. (2020b)*. Words that are more well-connected in semantic networks are advantaged in immediate serial recall, across serial position and independently of frequency.* Manuscript in preparation.

[CR34] Nelson, D. L., Bennett, D. J., Gee, N. R., Schreiber, T. A., & McKinney, V. M. (1993). Implicit memory: Effects of network size and interconnectivity on cued recall. *Journal of Experimental Psychology: Learning, Memory, and Cognition, 19*, 747–764. 10.1037/0278-7393.19.4.747 10.1037//0278-7393.19.4.747 8345322

[CR35] Nelson DL, McEvoy CL, Schreiber TA (2004). The University of South Florida Free Association, Rhyme, and Word Fragment Norms. Behavior Research Methods, Instruments, & Computers.

[CR36] Nelson DL, Schreiber TA (1992). Word concreteness and word structure as independent determinants of recall. Journal of Memory and Language.

[CR37] Palan S, Schitter C (2018). Prolific.ac—A subject pool for online experiments. Journal of Behavioural and Experimental Finance.

[CR38] Qiu M, Johns BT (2020). Semantic diversity in paired-associate learning: Further evidence for the information accumulation perspective of cognitive aging. Psychonomic Bulletin & Review.

[CR39] R Core Team. (2019). R: A language and environment for statistical computing [Computer software]. Vienna, Austria: R Foundation for Statistical Computing. Retrieved from https://www.R-project.org/

[CR40] Rabbitt P, Lowe C (2000). Patterns of cognitive ageing. Psychological Research.

[CR41] Roy, B. C., Frank, M. C., DeCamp, P., Miller, M., & Roy, D. (2015). Predicting the birth of a spoken word. Proceedings of the National Academy of Sciences, 112(41), 12663–12668. 10.1073/pnas.1419773112 10.1073/pnas.1419773112 PMC4611597 26392523

[CR42] Siew CSQ (2019). Spreadr: An R package to simulate spreading activation in a network. Behavior Research Methods.

[CR43] Siew CSQ, Vitevitch MS (2016). Spoken word recognition and serial recall of words from components in the phonological network. Journal of Experimental Psychology: Learning, Memory, and Cognition.

[CR44] Siew, C. S. Q., & Vitevitch, M. S. (in press). An investigation of network growth principles in the phonological language network. *Journal of Experimental Psychology: General*. 10.1037/xge0000876 10.1037/xge0000876 32584127

[CR45] Siew CSQ, Wulff DU, Beckage NM, Kenett YN (2019). Cognitive network science: A review of research on cognition through the lens of network representations, processes, and dynamics. Complexity.

[CR46] Steyvers M, Tenenbaum JB (2005). The large-scale structure of semantic networks: Statistical analysis and a model of semantic growth. Cognitive Science.

[CR47] Tse CS, Altarriba J (2007). Testing the associative-link hypothesis in immediate serial recall: Evidence from word frequency and word imageability effects. Memory.

[CR48] Vitevitch MS, Stamer MK (2006). The curious case of competition in Spanish speech production. Language and Cognitive Processes.

[CR49] Zaretsky HH, Halberstam JL (1968). Effects of aging, brain-damage, and associative strength on paired-associate learning and relearning. The Journal of Genetic Psychology.

